# Atomic-resolution three-dimensional hydration structures on a heterogeneously charged surface

**DOI:** 10.1038/s41467-017-01896-4

**Published:** 2017-12-13

**Authors:** Kenichi Umeda, Lidija Zivanovic, Kei Kobayashi, Juha Ritala, Hiroaki Kominami, Peter Spijker, Adam S. Foster, Hirofumi Yamada

**Affiliations:** 1 Department of Electronic Science and Engineering, Kyoto University, Katsura, Nishikyo, Kyoto 615-8510 Japan; 2 Department of Advanced Material Science, The University of Tokyo, Kashiwa, Chiba 277-8561 Japan; 3 COMP Centre of Excellence, Department of Applied Physics, Aalto University, Helsinki, FI-00076 Finland; 4 The Hakubi Center for Advanced Research, Kyoto University, Katsura, Nishikyo, Kyoto 615-8520 Japan; 5 Division of Electrical Engineering and Computer Science, Kanazawa University, Kanazawa, 920-1192 Japan

## Abstract

Local hydration structures at the solid–liquid interface around boundary edges on heterostructures are key to an atomic-level understanding of various physical, chemical and biological processes. Recently, we succeeded in visualising atomic-scale three-dimensional hydration structures by using ultra-low noise frequency-modulation atomic force microscopy. However, the time-consuming three-dimensional-map measurements on uneven heterogeneous surfaces have not been achieved due to experimental difficulties, to the best of our knowledge. Here, we report the local hydration structures formed on a heterogeneously charged phyllosilicate surface using a recently established fast and nondestructive acquisition protocol. We discover intermediate regions formed at step edges of the charged surface. By combining with molecular dynamics simulations, we reveal that the distinct structural hydrations are hard to observe in these regions, unlike the charged surface regions, possibly due to the depletion of ions at the edges. Our methodology and findings could be crucial for the exploration of further functionalities.

## Abstract (web summary)

Local hydration structures at solid-liquid interfaces are important in catalytic, electrochemical, and biological processes. Here, the authors demonstrate atomic-scale 3D hydration structures around the boundary on a heterogeneous mineral surface using atomic force microscopy experiments and molecular dynamics simulations.

## Introduction

Along with growing concern over energy and environmental issues, functional materials and devices utilising the catalytic, electrochemical and even biological processes at the solid–liquid interface have gained great attention^1–7^. Heterostructures, nano-clusters, edges and defects are particularly crucial in these processes, because these structures often amplify the reaction efficiencies due to quantum effects, low-coordinated atoms or concentrated electric fields^2–4, 7^.

Since they are always accompanied by local hydration changes reflecting the local structural and chemical changes of adsorption/surface species, understanding the local hydration structures at such reactive places is also essential. The hydration structures in complicated biological crystal samples have been extensively studied by X-ray/neutron crystallography, nuclear magnetic resonance and cryoelecton microscopy^8, 9^. However, all of these measurements require samples in a crystalline state, of which the hydrations at such local structures are averaged out and also often differ from those in liquid environments. Although the hydration structures in liquid environments have been extensively studied by X-ray/neutron reflectivity^10^ and surface force measurements^11^, their lateral structure has hardly ever been observed.

Recently, we have opened the door to atomic-scale three-dimensional (3D) mapping of the local hydration structures by ultra-low noise frequency-modulation atomic force microscopy (FM-AFM)^12, 13^, which has been applied to various homogeneous samples^12–18^. Moreover, with the support from theoretical molecular dynamics (MD) simulations, deeper molecular detail has been achieved^17, 19–23^. However, the time-consuming atomic-scale 3D hydration measurements on the uneven heterogeneous surfaces have never been achieved, to the best of our knowledge, because these experiments are extremely difficult without any deformation of the surface structures by the tip. Therefore, no one has yet observed atomic-scale local hydration structures between surfaces of different compositions as far as we know. Furthermore, the relationship between the local hydrations and surface properties on heterogeneous systems was theoretically discussed^24^, but validation against experiments is also necessary. Previously, we established a fast and nondestructive acquisition protocol of 3D map measurements on uneven heterogeneous DNA samples without damaging the molecules^25^ (see ‘Methods’ section for details).

In this study, we show the local hydration structure around a domain boundary between heterogeneous areas with different structures and charges on a phyllosilicate surface. Molecular-scale hydration structures on the terraces are visualised by 3D FM-AFM in aqueous solution, which are reproduced by the MD simulations. The simulations show different ion adsorption behaviours on oppositely charged terraces, which are reflected in the long-range electrostatic force in the experiment. We reveal the intermediate regions near the step edge on the positively charged terrace by 3D FM-AFM, presumably caused by the ion depletion near the step edge, which is predicted by the MD simulations.

## Results

### Structure of a heterogeneously charged surface

**Fig. 1:**
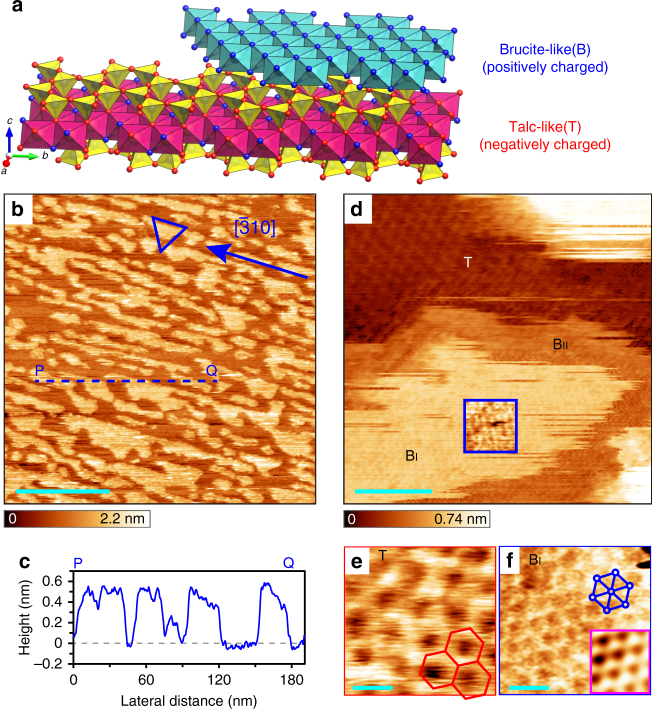
FM-AFM images of clinochlore. **a** Crystal structure of clinochlore crystal, which is composed of negatively charged talc-like (T) and positively charged brucite-like (B) layers. The figure was drawn using VESTA^42^. **b** Topographic image of the clinochlore (001) surface. The triangle shows the characteristic shapes due to the partial dissolution of the B layer^29^. **c** Cross-sectional profile along the P–Q line indicated in **b**. **d** Atomic-resolution image of the clinochlore (001) surface showing that the B island has two regions (B_I_ and B_II_) with different heights. In the blue square, the contrast was enhanced and smoothed by a Gaussian filter. **e**, **f** Magnified atomic-resolution images taken on the T region (**e**), and B_I_ region (**f**), with the inset of an FFT-filtered image. All of the images were acquired with ∆*f* ~ + 300 Hz. Scale bars: 100 nm (**b**), 3 nm (**d**), and 0.5 nm (**e**, **f**)

Clinochlore, (Mg,Fe)_5_Al(Si_3_Al)O_10_(OH)_8_, belongs to the chlorite group of phyllosilicate materials, which are ubiquitous minerals existing over wide temperature and pressure ranges^26–29^ (see Supplementary Methods for determination of the composition and orientation of the crystal used in this study). It is composed of alternating structures of a tetrahedral–octahedral–tetrahedral (2:1) negatively charged talc-like (T) layer of [(Mg,Fe)_3_(Si_3_Al)O_10_(OH)_2_]^–^ and the positively charged brucite-like (B) layer of [(Mg,Fe)_2_Al(OH)_6_]^+^ (Fig. 1a and Supplementary Movie 1). Note that each layer is equivalent to phlogopite mica or hydrotalcite excluding the intercalating ions^24^. Upon cleavage of the crystal, this unique character of clinochlore allows us to investigate the step edges where the T and B layers alternate in great detail with both the 3D FM-AFM and MD simulations.

After cleavage of the sample, we obtained a large-scale topographic image of the clinochlore (001) surface in a 100 mM KCl aqueous solution (Fig. 1b). In this image, we see island structures with stripe-like patterns running along the [${\bar{3}}$10] direction, with the typical size of 100 × 10 nm^2^. From the cross-sectional line profile along P–Q (Fig. 1c), we deduced the island heights to be about 0.5 nm, the thickness of the B monolayer^27, 28^, meaning that the higher islands are the B layer and the other regions are the T layer. Note that some islands in Fig. 1b have characteristic triangular shapes and step directions similar to the side of the drawn blue triangle, as previously reported^29, 30^.

In order to investigate the clinochlore island structure, we obtained an atomic-scale topographic image (Fig. 1d). On the T layer, atomic contrast is clearly visible, while the B layer seems to be composed of central and peripheral regions, indicated as B_I_ and B_II_, respectively. The B_II_ region has a lower height of 0.3 nm instead of 0.5 nm for the B_I_ region. Figure 1e, f show enlarged atomic resolution images of the T and B_I_ regions, respectively. We observed honeycomb-like patterns with a 0.53-nm spacing on the T regions and a hexagonal lattice with a 0.31-nm spacing on the B_I_ regions. In the B_II_ regions, a similar hexagonal pattern was observed, but was less clear than on B_I_ (Supplementary Fig. 1).

### Comparison between the experimental and theoretical data

**Fig. 2:**
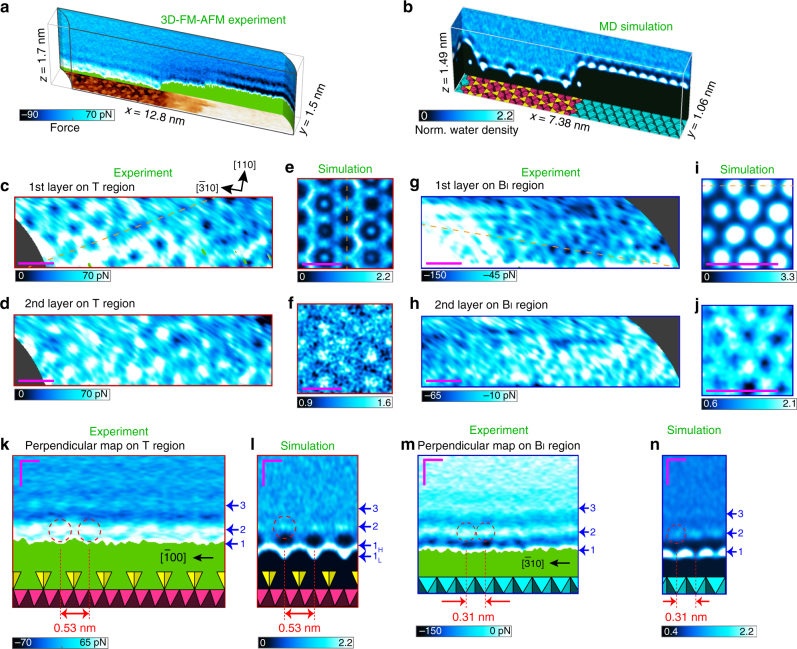
Comparison of 3D FM-AFM and MD results on each terrace region. **a**, **b** Representations of 3D force map (**a**) and MD simulation (**b**) on the clinochlore (001) surface. **c**, **d**, **g**, **h** Lateral 2D force maps measured at the tip heights presumably corresponding to the first (**c**) and second (**d**) layers on the T region, and the first (**g**) and second (**h**) layers on the B region. **e**, **f**, **i**, **j** Theoretical lateral 2D-normalised water (oxygen) density maps of the first higher (**e**) and second layers (**f**) on the T region, and the first (**i**) and second layers (**j**) on the B region. **k**, **m** Perpendicular 2D force maps along the broken lines in **c** on the T region (**k**), and **g** on the B_I_ region (**m**). **l**, **n** Theoretical perpendicular 2D-normalised water (oxygen) density maps along the broken lines in **e** on the T region (**l**), and **i** on the B region (**n**). The adsorbed first lower layer in the centre of the honeycomb and the first higher layer forming the honeycomb-like pattern are shown as 1_L_ and 1_H_, respectively. The red broken circles indicate the periodic hydration structures. Zero-distances in the simulations are defined as the centres of the oxygen atoms of each surface. Scale bars: 0.8 nm (**c**–**j**) and 0.3 nm (**k**–**n**)

In Fig. 2a, b, we show representations of the 3D force map obtained across an area including the T, B_II_ and B_I_ regions (Supplementary Movie 2) and the equivalent MD simulation (Supplementary Note 1 and Supplementary Movie 3), respectively. In order to better understand the nature of the B_II_ region, we first focus in detail on the hydration structures found on the B_I_ and T regions. Previous studies have already shown that the force profiles or maps obtained by AFM gave good qualitative agreement with the density distributions of the water molecules determined by the X-ray reflectivity or predicted by the MD simulations^13, 14, 17, 23, 31–35^. Specifically, it has been reported in some papers that AFM force maps were well reproduced by free-energy calculations^17, 32^. We first analysed the hydration layers on the T region in the same manner as in ref. ^13^, where we assumed that the hydration force maxima correspond to the density maxima. For the T region, we could clearly distinguish two hydration layers. In Fig. 2c, the honeycomb-like pattern of the first hydration layer (closest to the surface) as observed in the AFM experiments is shown, and a similar pattern (Fig. 2e) is seen in the 2D water density distribution of the first higher layer. The second hydration layer above the T region follows much more like a dot-like pattern in both the experiment and simulation (Fig. 2d, f). We can see highly localised spots in the honeycomb hollows in the 2D water density distribution of the first higher layer (Fig. 2e). These dots are seen only in some honeycomb hollows because of the limited simulation time and they correspond to the tails of the adsorbed first lower layer, mainly contributed by the water molecules adsorbed in the honeycomb hollow. These are related to counter cations resident at the sites, but would be averaged out, and appear much fainter, if the simulations could be run long enough to obtain full sampling of the ion mobility. In the case of the B_I_ region, atomic-scale patterns with the same lattice constant are observed in both the experiment (Fig. 2g, h) and simulation (Fig. 2i, j). While the 2D water density distributions show bright and dark dot patterns for the first (Fig. 2i) and the second (Fig. 2j) hydration layers, respectively, the patterns observed in the experiment are somewhat ambiguous. It is difficult to judge if they are the same as those predicted from the simulation or even inverted because the contrast varies as a function of the tip height and conditions^36^. Since the lattice spacing of the B layer is smaller than that of the T layer and comparable to the size of the water molecule, it is plausible that the hydrated water molecules other than that on the tip apex play a role in the contrast formation and cause the patterns to be ambiguous or possibly inverted. As we show later, the anions form a honeycomb-like pattern at the same height of the first hydration layer on the B layer, which is the inverted pattern of the water (oxygen) distribution and may play a role in this observation.

From both the experimental and theoretical data, we extracted force and density maps perpendicular to the clinochlore surface along the lines indicated in Fig. 2c, e, g, i. On each of these four maps, we can identify the respective lateral spacing, which is 0.53 nm for the T region (Fig. 2k, l) and 0.31 nm for the B_I_ region (Fig. 2m, n).

From the discussion above, we found that the apparent surfaces (tip trajectories) are the first layers instead of the bare mineral surface, and the molecular-scale patterns observed in the experiment were in good agreement with the 2D water density distributions in the first and second hydration layers. It should be mentioned that when we set the threshold values higher, the tip change was often induced by a strong interaction force and the continuity of the consecutively recorded force curves was deteriorated. These results demonstrate that the combination of liquid-3D FM-AFM and MD simulation is useful for illuminating the details of 3D water distributions.

### Quantitative analysis of force curves

**Fig. 3:**
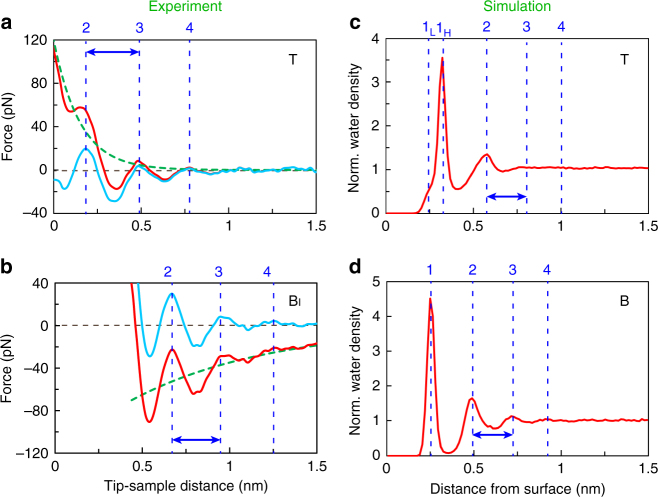
Comparison of force curves and density profiles. **a**, **b** Average force profiles on the T (**a**) and B_I_ (**b**) regions extracted from 3D force map (red curves). The broken green and solid light-blue curves are the fitted background force and subtracted force curves, respectively. The vertical blue broken lines represent the force maxima in the subtracted curves. **c**, **d** Normalised water (oxygen) and ion density profiles calculated by MD simulation on the T (**c**) and B (**d**) regions

For more quantitative analysis, we also constructed averaged force curves (measured at 200 randomly selected pixels) for both the T and B_I_ regions from the experiment (Fig. 3a, b). While the similar oscillatory features are obvious in both cases, the repulsive and attractive background forces are seen on the T and B_I_ regions, respectively. We fitted these background forces by exponential functions with decay distances of 0.15 and 0.9 nm for the T and B_I_ regions, respectively. The decay distance of 0.9 nm on the B_I_ region is almost equivalent to the Debye length in the 100 mM KCl solution, meaning that this force component is likely the long-range electric double-layer force^25^. The reason why this force was hardly observed on the T region can be explained by the fact that most of the surface-negative charges on the T layer are neutralised by the coadsorption of the proton and cation in the solution of pH 5.7^37^. This strong adsorption of cations on the T layer is also seen in our simulations, which will be shown later. For this reason, while the structural charge density of the T (–) and B (+) layers is the same, ±0.32 C/m^2^, the apparent surface charge densities are different, –0.015 and +0.32 C/m^2^, respectively^37, 38^. We estimated that the surface charge density on the B_II_ region was +0.05 C/m^2^ (Supplementary Note 2), which was significantly lower than that on the B_I_ region due to the edge effect as discussed later. For the short-range force observed on the T regions, we consider that it was possibly due to the relaxation of the tip/sample atoms, as previous theoretical studies on the interactions between the positive/negative tip on the positive/negative ions of calcite in solutions showed different tip relaxation behaviours depending on the tip/sample polarities^17, 36^. We also consider that the short-range repulsive force could be due to strongly bound cations in the hollow sites, as we also observed a similar short-range repulsive force on muscovite mica in a high-salt solution^13^. Since we cannot determine the tip configuration during the experiment, identifying the role of the tip would require a very extensive set of simulations including free-energy calculations using various tip/sample models and more experiments to identify the origin of the short-range force. This is an ongoing challenge for the field, but is beyond the scope of the current work.

We retrieved the normalised water density curves (averaged over the entire surfaces) from the MD simulations (Fig. 3c, d). When comparing the hydration peak distances, we consistently observed that there was no intrinsic difference in the hydration layer spacing for either the T or B_I_ regions; namely, 0.31 and 0.30 nm in the experiments, and 0.25 and 0.24 nm in the simulation for the T and B_I_ regions, respectively (see arrows in Fig. 3). Although the experimental forces and theoretical water densities have many features in common, in principle, we cannot compare them directly^17^. To overcome this problem, we also converted the densities to force by the recently proposed solvent tip approximation model^39^, and we obtained consistent results regarding the terrace regions (Supplementary Note 3).

### Intermediate region around the step edge

**Fig. 4:**
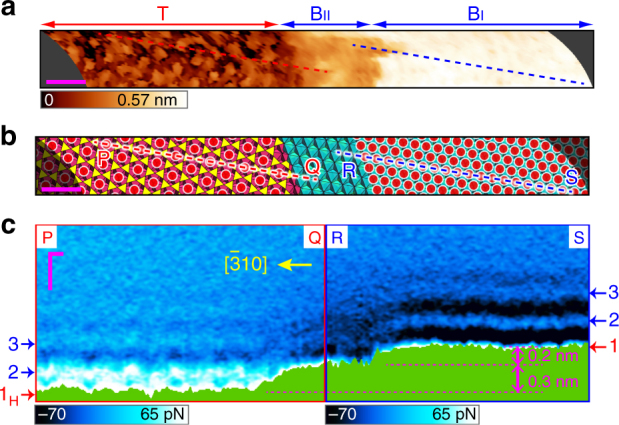
Experimental local hydrations around the step edge. **a**, **b** Topographic image (**a**) and structural model (**b**) of the clinochlore (001) surface reconstructed from 3D Δ*f* data (Δ*f* = 290 Hz). Red dots represent the adsorbed water molecules on the first layers on the T and B_I_ regions. **c** Perpendicular 2D force maps extracted from the 3D force data along the broken lines P–Q and R–S in **a**. Scale bars: 1 nm (**a**, **b**) and 0.3 nm (**c**)

From the 3D FM-AFM data, we reconstructed a topographic image of the clinochlore (001) surface (Fig. 4a), whose model is shown in Fig. 4b. Along the broken lines P–Q and R–S (both running in the [${\bar{3}}$10] direction), we extracted the 2D force maps (Fig. 4c). On the B_II_ region, we did not observe the dot-like patterns, which was consistent with the result that atomic-resolution imaging on the B_II_ region was less clear than that on the B_I_ region. As the apparent height of the B_II_ region was about 0.3 nm, we conclude that the tip was scanned over the first layers on the T and B_I_ regions, giving the atomic resolution on both the regions, while the tip was directly scanned over the solid surface in the B_II_ region.

## Discussion

**Fig. 5:**
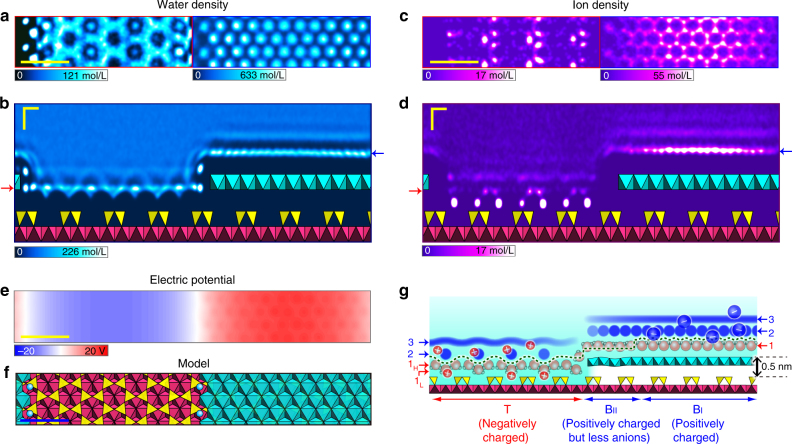
Theoretical local hydrations and ions around the step edge. **a**, **c** Lateral water (oxygen) (**a**) and ion (**c**) density maps from the MD simulation in the first layers on the T and B regions. **b**, **d** Perpendicular water (**b**) and ion (**d**) density maps averaged along the [001] direction, where the arrows indicate the heights of the lateral water density maps in **a** and **c**, respectively. **e** Electric potential of 0.1 nm above the outermost oxygen atoms of the B region (showing a zero potential at the step edge, in white). **f** Structural model used in the MD simulation. **g** Schematic illustration of the cross section of the clinochlore (001) surface along the dashed lines in Fig. 4a, b. The green dashed curve shows the apparent surface (trajectory of the tip scan during imaging topography). Scale bars: 1.0 nm (**a**, **c**, **e**) and 0.3 nm (**b**, **d**)

We show 2D theoretical water (oxygen) and ion density maps in Fig. 5a, b, which are presented with the unit of mol/L and not normalised to the bulk density. Note that we used a 1 M solution in the initial state due to time and size constraints (see Supplementary Fig. 6c for the snapshot), which had a ten times higher ion concentration than the experimental condition. Under this condition, the water density is still orders of magnitude larger than the ion density even in the vicinity of the charged surfaces. We consider that the ions were moving around during the millisecond timescale of the AFM measurements, and thus that the experimentally observed hydration forces and their contrast patterns in the 2D maps mainly originate from the water density distribution. This is supported by repeated observations of the common similarities between experimental force and simulated water/force on several other surfaces^13, 14, 17, 21, 23, 31–35^. A recent paper on FM-AFM measurements of the hydration structures of calcium fluorite in ultra-pure water and supersaturated solution has shown that the adsorbed ions could give a slight modulation in the oscillatory force profiles, but they did not change the overall features^33^. Actually, we also observed the hydration structures on muscovite mica in ultra-pure water as well as in high-salt solutions (Supplementary Note 4). We do not deny the role of ions in the AFM measurement, but their effects are limited. For the T region, Fig. 5d shows that the innermost cations (Na^+^) are located closer to the surface than the oxygens of the water molecules. As already mentioned, the short-range repulsive force observed on the T region could be because of these strongly bound cations in the hollow sites. On the other hand, for the B region, we see in Fig. 5d that the innermost anions are competing with the water oxygens at almost the same height, and they create the opposite patterns (see the right side of Fig. 5a, c). As previously mentioned, the ambiguous or possibly inverted 2D patterns observed on the B region could be due to the anions (Cl^–^). This result is consistent with the experimental result that we observed, the long-range electrostatic force on the B region but not on the T layer probably because most of the negative charges on the T region are screened by the cations inside the hydration layer. Finally, it should also be noted that several researchers have resolved individual adsorbed ions by AFM if they strongly bound on the surfaces with the timescale longer than the AFM measurements^22, 40^.

The MD simulations also suggest a reason why no clear hydration structures could be observed in the B_II_ region. Around a step edge, the water densities in the first layers (top view, Fig. 5a, and side view, Fig. 5b) indeed show an increase, while the ion (cation and anion) densities (top view, Fig. 5c, and side view, Fig. 5d) are decreased. The orientations of the water molecules around the step edges were comparable to those on the terrace, meaning that this depletion of ions is a possible candidate for the indistinct hydration structure. It has been experimentally shown that a high ion concentration often enhances the image contrast of the hydration structure^12–16^, which can be explained by the reduction in the diffusion coefficient and the increase in the residence time of the water molecules^24, 41^.

In order to discuss the origin of the depletion, we calculated the electric potential as shown in Fig. 5e (see Supplementary Methods for the details). As a result, we found a neutral area around the step edge, and the potential on the B layer was reduced in the vicinity of the step edges. In the case of an electrified metal, where the boundary condition is the constant-potential, the electric fields and ion concentration around edge structures are enhanced due to the edge effect^3^. On the other hand, in the case of a fully charged system such as the B layer, where the boundary condition is a constant-charge, the electric fields around the edges are the same as those on the flat surfaces, while the surface potentials and ion concentration around the edges significantly decrease. The reduced surface potential causes the depletion of ions, while the constant electric field maintains the same hydration structure as that on the terrace. Increasing the ion concentration reduced the width of the B_II_ region (Supplementary Fig. 2), which supports this explanation.

A schematic illustration of the cross section of the clinochlore (001) step deduced from the experiments and simulations is shown in Fig. 5g. We explicitly draw the water molecules in the first layers and use blue patterns as the upper hydration layers observed by liquid-3D FM-AFM. We only observed the multiple hydration layers on the T and B_I_ regions, but barely observed any layers on the B_II_ region although there are hydration layers on the B_II_ region as well. Our results clearly show that the liquid-3D FM-AFM can distinguish the local hydration structures reflecting different surface properties even at the domain boundaries.

In summary, we performed the 3D FM-AFM experiment and MD simulations on the clinochlore (001) surface in aqueous solution. By comparing these results, we conclude that lattice periodicities are reflected in the intrinsic lateral water distributions, which are experimentally observed as a laterally periodic hydration structure. Meanwhile, the surface charge difference is reflected in the adsorption behaviour of the ion species, which are experimentally observed as the long-range background force. We also discovered intermediate regions at the step edges of the B islands, which likely originate from ion depletion by the edge effect, although the stability of different step morphologies could also play a role.

This work is, to the best of our knowledge, the first demonstration of the 3D FM-AFM measurements on an inhomogeneous mineral surface since 3D FM-AFM has so far been applied mainly to atomically flat homogeneous mineral surfaces^12–15, 17, 32–34^. This was quite challenging because it has step-and-terrace structures and the oppositely charged areas. However, we developed a fast and nondestructive acquisition protocol by disabling the constant frequency shift feedback and setting the frequency threshold instead, which enabled us to acquire the 3D map on an uneven heterogeneous mineral surface. We consider this as an important milestone in the development of the FM-AFM/MD method, and we hope that the methodology, once established, could be applicable for the hydration structures of more complicated systems such as catalysis, electrochemistry and even biological molecules including proteins and nucleic acids in the future. Here, we also remark that the clinochlore (001) surface can also serve as an experimental platform for exploring the biological processes because it can be used for patterning^27^ or suspension (Supplementary Note 5) of biological molecules. Since FM-AFM could give some information on the outer hydration shells surrounding biomolecules in vivo, we consider that it is useful as a complementary tool to the conventional techniques that mainly give the information on the distribution of the water molecules inside the biomolecules.

## Methods

### FM-AFM setup

We used a customised commercial AFM head (Shimadzu: SPM-9600) with a homebuilt digital PXI controller (National Instruments: NI PXI-8196) based on a high-speed field-programmable gate array (FPGA) board (National Instruments: NI PXI-7833R) programmed by LabVIEW (National Instruments) and a homebuilt FM detector circuit^43^. In order to achieve quantitative and reproducible force measurements in a liquid environment, we employed a photothermal excitation setup^44^. We used a rectangular cantilever with a gold backside coating (Nanosensors: PPP-NCHAuD), whose nominal spring constant (*k*_*z*_) was 42 N/m. The *k*_*z*_ of the cantilevers used in the experiments was determined to be 35 N/m by Sader’s method^45^. The resonance frequency of the cantilever (*f*_0_) was 131 kHz in a 100 mM KCl solution. Immediately prior to each experiment, organic contaminations on the tip were removed by irradiating the tip using a UV-ozone cleaner (Filgen: UV253) for a few hours.

The measurement was performed in 100 mM potassium chloride aqueous solutions (KCl, 99.5% purity, Wako Pure Chemical Industries, Ltd.) whose Debye length is 0.97 nm at 298 K. This reagent was used without further purification and any pH regulation. The aqueous solution was slightly acidified to a pH value of around 5.7 due to the dissolved CO_2_ gas. All the experiments are conducted in a temperature-regulated enclosure (Mitsubishi Electric Engineering Company, Ltd.: CN-40A), which can maintain a constant temperature of 298 ± 0.1 K and thus reduce the influence of thermal drift by the AFM head.

### 3D FM-AFM measurement

The 3D map measurement on an uneven heterogeneous surface was realised by the real-time monitoring^13, 25^, instead of a time-averaging monitoring^12^, of the frequency shift, which was implemented by a high-speed FPGA board. The signal-to-noise ratio and the acquisition time of the 3D map measurements are in a trade-off relation. When the scanning speed is reduced, the risk of surface deformation decreases, but the thermal drift increases. Conversely, when the scanning speed is increased, the thermal drift is reduced, but the surface deformation can occur. In order to keep a high-enough signal-to-noise ratio while suppressing the thermal drift, we need several improvements to the FM-AFM experiment system as follows.

A 3D Δ*f* map with dimensions of 12.8 × 1.5 × 1.7 nm^3^ (256 × 15 × 173 pixels) was acquired by collecting perpendicular 2D Δ*f* maps (XZ slices). During the 2D Δ*f* map acquisition, the frequency shift vs. distance curves (1D Δ*f* curves) were recorded by translating the tip towards the sample using a sawtooth waveform signal of 22 Hz, which corresponds to a tip velocity of about 40 nm/s. The approach was immediately stopped when the frequency shift signal reached a predetermined threshold value, and then the tip was retracted to the original position. Proper setting of the threshold value minimises the possibility of the tip change as well as the sample damage, and thereby secures the continuity of the consecutively recorded force curves. By omitting the retraction curve measurement, the total acquisition time can be reduced by almost half to 1.5 min. Although it has been demonstrated that a 3D Δ*f* map can be collected by employing a sinusoidal tip motion with a slow constant frequency shift feedback^12, 14, 16–18, 32, 33^, it is not suitable for acquiring a 3D Δ*f* map on an uneven heterogeneous surface, where large variations in the topographic height and long-range electric double-layer force are expected during the data acquisition.

In ultra-high vacuum (UHV), the atom-tracking technique is often used for suppressing the thermal drift^46^. However, in a liquid, it is difficult to implement because the thermal drift is nonlinear and the tip change often occurs due to the higher interaction force than that in the UHV. In order to solve this problem, we used a real-time feedforward system. Additionally, an automatic post-processing programme is also crucial for the efficient time-consuming 3D map measurements.

As described above, the photothermal excitation setup^44^ is essential for stabilising the uncontrollable fluctuation of the resonance frequency. The preparation method for the tip and sample is also essential for high-resolution 3D map imaging. Any organic contaminants on the tip should be removed by a UV-ozone cleaner immediately before each experiment for avoiding unstable tip conditions. A droplet of the solution should be placed on the sample as soon as possible after the cleavage of the sample for avoiding any contamination. Since an adhesive for fixing the sample to a magnetised stainless-steel plate often contaminates the solution, a larger sample size should be used in order to cover the entire stainless-steel plate.

### Post-processing of 3D FM-AFM data

We established an automatic post-processing programme based on Visual Studio (Microsoft), which is essential for efficient analysis of the large-size 3D map data. First, the 3D Δ*f* data were smoothed by using a Gaussian filter with a standard deviation of 0.034 nm (*x* and *y*) and 0.011 nm (*z*), and the 3D force data were obtained by converting each 2D Δ*f* map to a 2D force map using Sader’s method^47^. Since the background offset of the 1D curves in the 2D map contained fluctuations, they were smoothed using a Gaussian filter with a standard deviation of 1.23 nm before and after the conversion. Second, the resolution of the 3D data was increased to 1024 × 113 × 346 pixels using a Lanczos interpolation filter of an order of 3 (Lanczos-3)^48^. Third, the background offset in each force curve was corrected using an analytical formula based on the electric double-layer theory^25^. Note that the force curve in Fig. 3b has a non-zero value in the farthest part because of the offset correction. Finally, the linear and nonlinear lateral drifts were corrected such that the lattice constants in the 2D lateral maps match those in the literature^49^. For the 2D force maps presented in Figs. 2k, m and 4c, the boundaries between the pixels with data (blue colour) and without data (green colour) were interpolated using the Lanczos-3 filter.

### MD simulations

All the MD simulations were performed using LAMMPS (Large-Scale Atomic/Molecular Massively Parallel Simulator) code^50^ under ambient conditions (300 K and 1 bar) using a Nosé–Hoover scheme and a time step of 1 fs. Along all the dimensions, periodic boundary conditions were employed. The surfaces were first optimised in vacuum in order to remove any unphysical interactions between overlapping atoms and then immersed into water. After water equilibration (for 25 ps), all atoms in the crystal were allowed to move, except for the Mg atoms in the central layer (in order to prevent drift of the entire system), and the system was equilibrated again for 25 ps. The target pressure was specified only along the *z* direction (to protect the crystal from lateral stress) allowing the simulation box to change during the dynamics, ensuring the proper water density in the bulk phase. The production MD runs with flat terraces were only performed for 10 ns, but it was increased to 15 ns for the systems with a clinochlore step present. This longer simulation time was necessary to allow ions in the solution to sufficiently diffuse in order to be able to reach the surface or step edge.

We obtained the crystal structure of clinochlore from the American Mineralogist Crystal Structure Database^49^. First, we simulated the hydration structures exposed on each region as shown in Fig. 2 (see Supplementary Fig. 6a, b for the snapshots). Based on the unit cell data, we constructed a simulation cell with lateral dimensions in the (001) plane of roughly 2.1 × 3.7 nm^2^. In the [001] direction (in terms of our Cartesian simulation cell, this is the *z* direction), we repeated sufficient layers of clinochlore to make sure that the internal part of the crystal is under no additional stress caused by the nearby free surfaces. A typical system contained ~2800 atoms for the clinochlore crystal and over 3000 water molecules.

Next, we simulated the hydration structures exposed on a step region as shown in Fig. 5 (see Supplementary Fig. 6c for the snapshot). In this case, we cleaved the surface along the (001) plane and removed one-half of the B layer to form the step edge running in the [100] direction, thus exposing the underlying layer as the lower terrace. In order to avoid a lateral dipole effect on the step edge, we created a larger system with the lateral dimension of 4.3 × 7.4 nm^2^. Step edges created this way were neutral. In order to investigate all possible edges that might appear in the real system, we modelled another step edge by cutting the B layer along the zigzag line (Supplementary Note 6). This way, we have created a step that matches triangular structures seen in experiments. This type of step edge was negatively charged due to the protruding oxygen atoms along the edge. In order to maintain a neutral system, an opposite triangle pit was introduced, consisting of exposed metal ions and therefore positively charged. In general, MD simulations showed that the triangular systems were not stable, with significant desorption of OH. Hence, we decided to focus on the stable (neutral) step edges in our simulations, but the results for the other steps are discussed in Supplementary Note 6. On either side of the clinochlore sample, water molecules were placed to solvate the system and to make sure that far away from the solid–liquid interfaces, water behaves as in the bulk. The system with the exposed step contained 4600 crystal atoms and ~13,000 water molecules.

All interactions between the atoms were described by two force fields. The CLAYFF force field^51^ was used to describe all interactions between the clinochlore atoms, water and ions present in solution. This force field has been successfully used in the past in many clay mineral MD simulations^24, 52, 53^. Although CLAYFF force field was developed to fully describe the properties of clay minerals, small modifications (<3%) of the partial charges were required in order to maintain an electrostatically neutral system for the surfaces and steps considered here (octahedral magnesium in the B layer +1.37 new/+1.36 CLAYFF; bridging oxygen with tetrahedral substitution in the T layer −1.16875/−1.1688 and hydroxyl oxygen in the B layer −0.9775/−0.95). However, these small deviations are consistent with charge-balancing approach in CLAYFF. As a water model in our simulations, we used the flexible TIP3P^54^ model. All parameters for this model were optimised to reproduce experimental thermodynamic and structural properties of liquid water as well as vibrational spectra. We also checked all simulations using the nonbonded three-body harmonic potential energy term for Mg–O–H interactions^55^ and modified charges^56^. These made no significant difference to our results on the standard step edge. However, the included nonbonded three-body term for Mg–O–H bending interactions significantly improved the stability of the triangle step structure (changing the charges did not), but not fully, as we still observed the desorption of some hydroxyl groups during simulations.

For visual inspection, VMD^57^ was used and most of the analysis was performed using the Python library MDAnalysis^58^. The 2D water density maps presented in Figs. 2 and 5 were created after smoothing the time-averaged 3D trajectory data using a Gaussian kernel filter with a standard deviation of 0.021 nm in all three directions and a linear interpolation.

### Data availability

The data that support the findings of this study are available from the corresponding authors upon reasonable request.

## Electronic supplementary material

Supplementary Information

Description of Additional Supplementary Files

Supplementary Movie 1

Supplementary Movie 2

Supplementary Movie 3
