# Author Correction: Atomic-resolution three-dimensional hydration structures on a heterogeneously charged surface

**DOI:** 10.1038/s41467-018-04401-7

**Published:** 2018-05-23

**Authors:** Kenichi Umeda, Lidija Zivanovic, Kei Kobayashi, Juha Ritala, Hiroaki Kominami, Peter Spijker, Adam S. Foster, Hirofumi Yamada

**Affiliations:** 1 Department of Electronic Science and Engineering, Kyoto University, Katsura, Nishikyo, Kyoto 615-8510 Japan; 2 Department of Advanced Material Science, The University of Tokyo, Kashiwa, Chiba 277-8561 Japan; 3 COMP Centre of Excellence, Department of Applied Physics, Aalto University, Helsinki, FI-00076 Finland; 4 The Hakubi Center for Advanced Research, Kyoto University, Katsura, Nishikyo, Kyoto 615-8520 Japan; 5 Division of Electrical Engineering and Computer Science, Kanazawa University, Kanazawa, 920-1192 Japan

Correction to: *Nature Communications* 10.1038/s41467-017-01896-4, published online: 13 Dec 2017

The original version of the Supplementary Information associated with this Article contained an error in Supplementary Figure 9e,f in which the *y*-axes were incorrectly labelled from ‘−40’ to ‘40’, rather than the correct ‘-400’ to ‘400’. The HTML has been updated to include a corrected version of the Supplementary Information.

