# Supplementary material for: Atomic-resolution three-dimensional hydration structures on a heterogeneously charged surface
Source: Nat Commun. 2017 Dec 13;8:2111. doi: 10.1038/s41467-017-01896-4 (PMC5727385; doi:10.1038/s41467-017-01896-4)
Supplement: Supplementary file 1 — Supplementary Information [file 41467_2017_1896_MOESM1_ESM.pdf]

Supplementary Figures

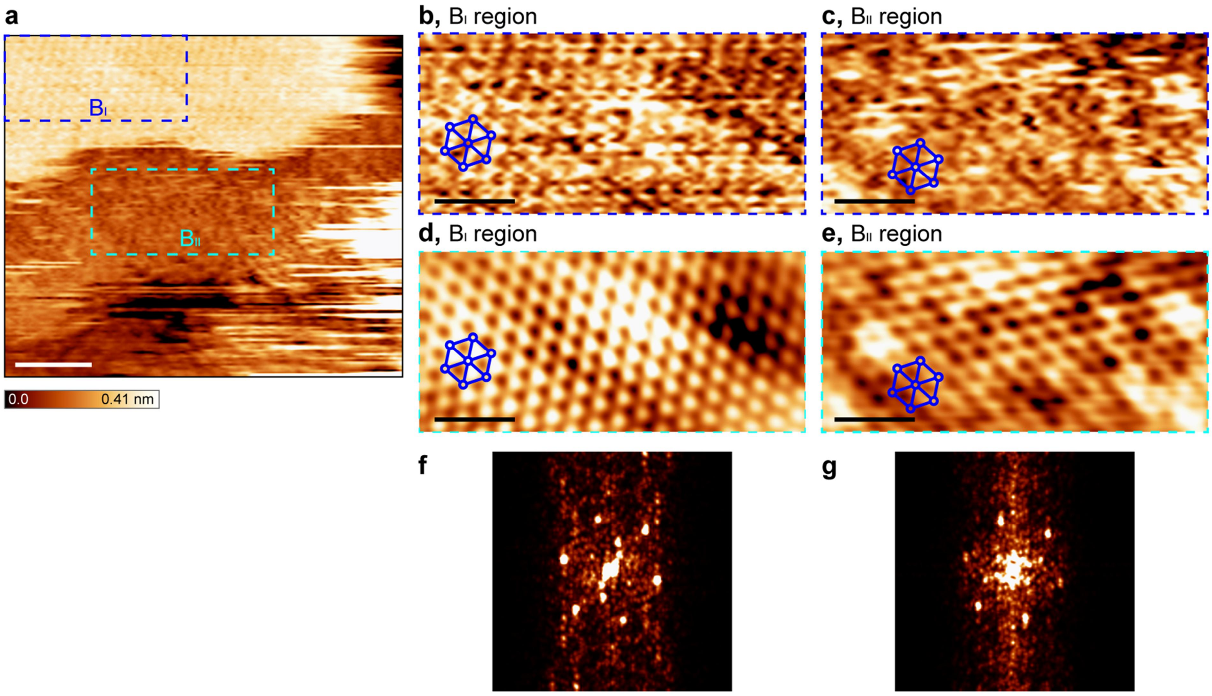

Supplementary Figure 1 | FM-AFM Images on brucite-like regions. a, Atomic-resolution image of the clinochlore (001) surface showing the B_I and B_{II} regions. **b**,**c,** Enlarged atomic-resolution images on the B_I (**b**) and B_{II} (**c**) regions extracted from the blue and light-blue broken line boxes in **a**, respectively. Atomic-scale contrast in the B_I region is clearer than that in the B_{II} region. **d**,**e,** FFT filtered images of **b** and **c**, respectively, showing a hexagonal lattice with a 0.31 nm spacing. **d**,**e,** FFT filtered images of **b** and **c**, respectively, showing an identical hexagonal lattice with a 0.31 nm spacing. **f**,**g,** Power spectrum images of **b** and **c**, respectively, showing an identical hexagonal pattern. Scale bars, 2 nm (**a**) and 1 nm (**b**-**e**).

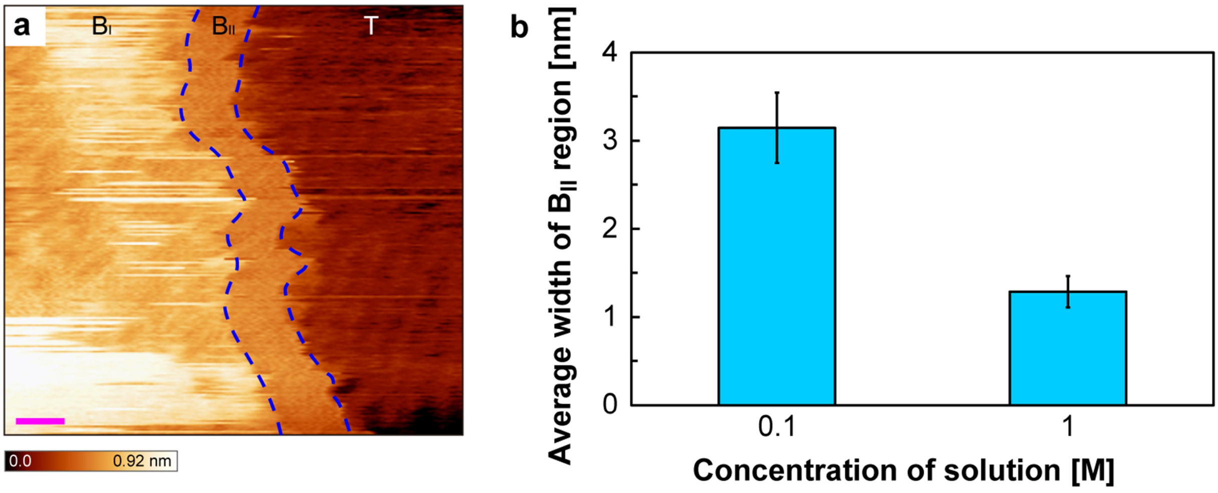

Supplementary Figure 2 | Dependence of the intermediate regions on the concentration of solution. a, Topographic image of the clinochlore (001) surface obtained in a 1 M KCl solution. The blue broken lines and arrow indicate the width of the intermediate B_{II} region. **b,** Statistical data of the relationship between the average width of the B_{II} region and the KCl solution concentration. The error bars represent the standard errors. The statistical analysis was conducted at 10 randomly selected locations. Scale bar, 1 nm (**a**).

Supplementary Methods

Clinochlore Crystal

High purity clinochlore single crystals, produced in Val Malenco, Sondrio Lombardy, Italy, were purchased from Hori Mineralogy Ltd. The chemical composition of our sample was explicitly determined by X-ray photoelectron spectroscopy (XPS, see Supplementary Fig. 3 and Supplementary Table 1) to be $(Mg_{5.14}Fe_{0.25}Al_{0.61})_{\Sigma 6.00}(Si_{3.30}Al_{0.70})_{\Sigma 4.00}O_{10}(OH)_8$. This results in the chemical composition of the talc-like (T) and brucite-like (B) layers to be $[(Mg,Fe)_{3.00}(Si_{3.30}Al_{0.70})O_{10}(OH)_2]^{-0.7}$ and $[(Mg,Fe)_{2.40}Al_{0.60}(OH)_6]^{+0.7}$, respectively. For reference, the result is shown with those of brucite and talc single crystals, which were produced in Cedar Hill Quarry, Lancaster County, Pennsylvania, USA and Argonaut Mine, Ludlow, Vermont, USA, respectively. The XPS analysis revealed that our sample contains higher amounts of Si and Mg and lower amounts of Al compared to that reported in Ref. 1.

The ideal structural charge density of the T (–) and B (+) layers are ±0.32 C/m^2. The XPS measurements resulted in an average charge density of our sample to be around ±0.22 C/m^2. Clinochlore is monoclinic (C2/m) with cell parameters of a = 0.5327 nm, b = 0.9227 nm, c = 1.4356 nm, α = 90.45°, β = 97.35°, and γ = 89.98°[2]. The crystallographic orientations of the clinochlore crystal were determined by the pole figure measurement (see Supplementary Fig. 4).

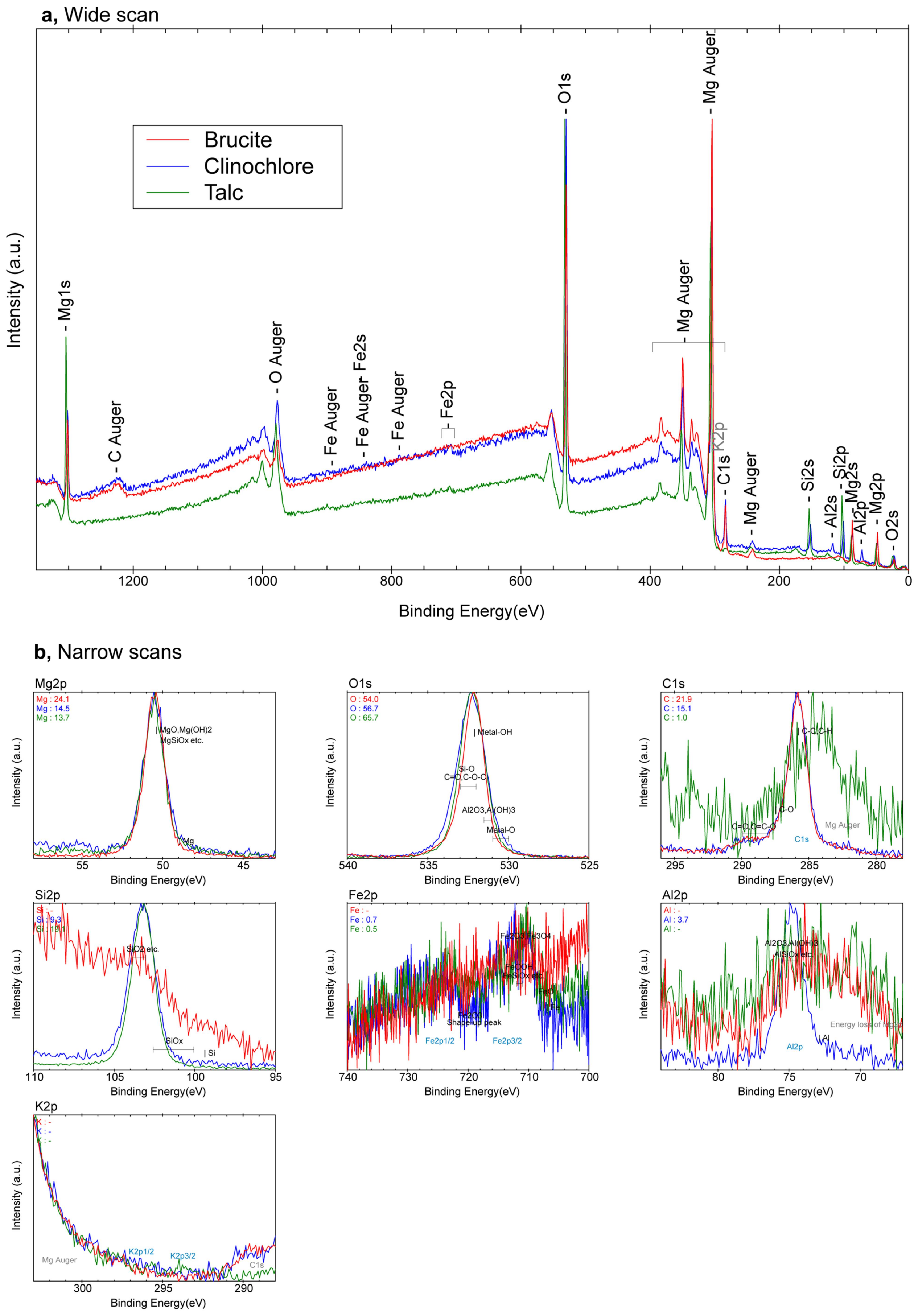

Supplementary Figure 3 | XPS spectra. a,b, XPS spectra of brucite, clinochlore and talc with wide (**a**) and narrow (**b**) scans.

Supplementary Table 1 | Analysis of XPS spectra. Surface compositions (atomic%) as determined from the XPS survey spectra, which contain ±1 atomic% errors.

	Mg	O	C	Si	Fe	Al	K
Brucite	24.1	54.0	21.9	0.0	0.0	0.0	0.0
Clinochlore	14.5	56.7	15.1	9.3	0.7	3.7	0.0
Talc	13.7	65.7	1.0	19.1	0.5	0.0	0.0

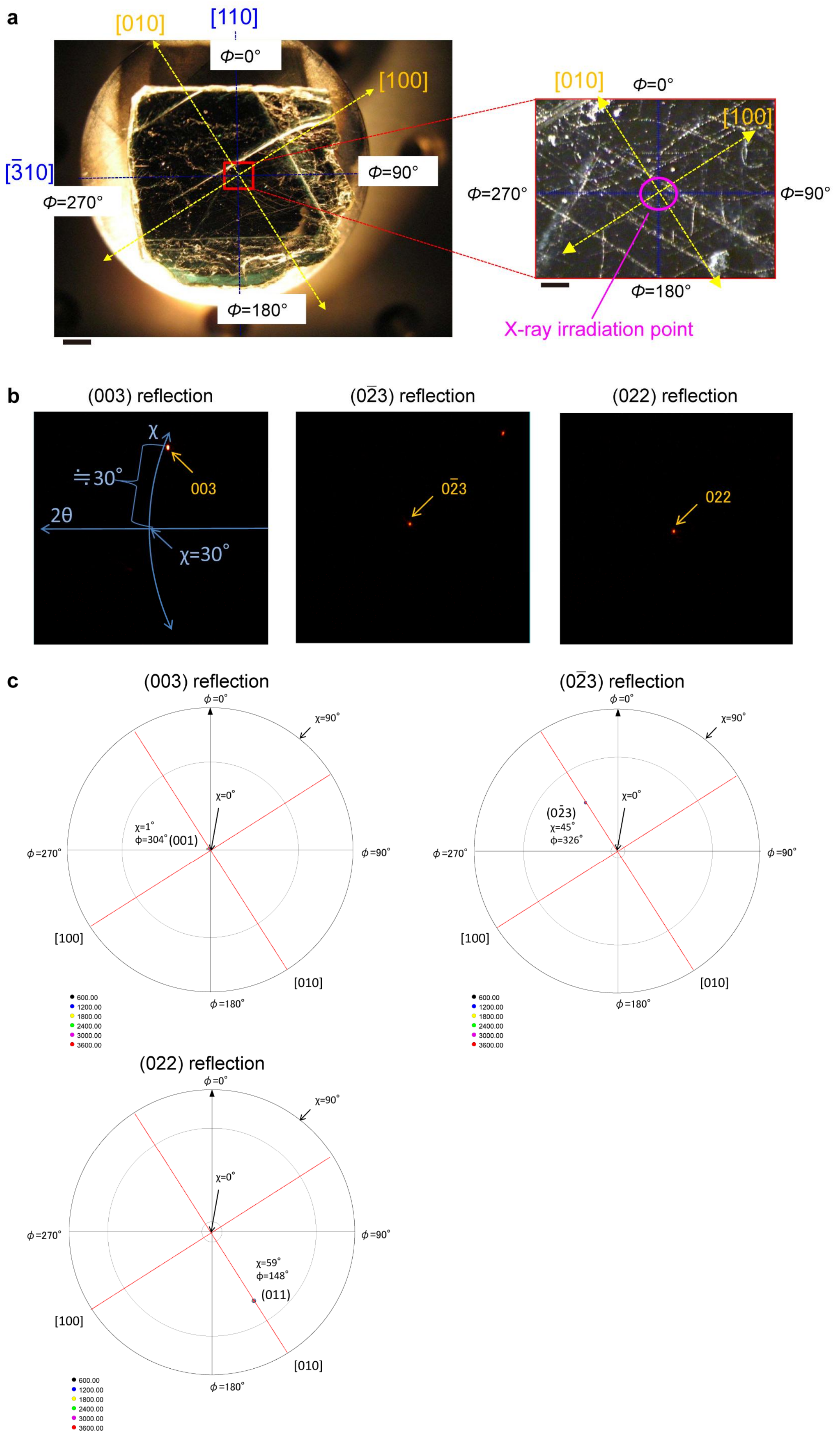

Supplementary Figure 4 | XRD pole figure measurement. a, Optical micrograph of a sample with indication of the X-ray irradiation point. **b,** X-ray diffraction patterns and **c,** pole figures obtained by XRD pole figure measurement. Scale bar, 200 μm (left in **a**) and 30 μm (right in **a**).

Calculation of Electric Potential

In the classical representation of atoms, the electrostatic interaction is defined by the point charges on the atoms. The potential energy and the force on a charged atom can be calculated from the electric potential generated by the other atoms in the system. There are other interactions affecting the dynamics of atoms besides the electrostatics, such as van der Waals interactions and the Pauli repulsion, but their range is shorter. Therefore, if the electric potential has a clear maximum or minimum at the length scale of several atoms, one can argue that the optimal minimum energy position of a charged atom exists in that region, as long as the position does not overlap with other atoms. We used this argument to justify why the solvated ions settle above the terraces, but avoid the step edge.

The long-range nature of the electrostatic interaction causes the direct summation of the Coulomb potentials of the point charges to converge very slowly as a function of distance. For the infinitely repeating simulation cell, this direct approach is thus extremely inefficient. Our method for solving the electric potential is based on the same idea that is used in the long-range part of the particle mesh Ewald method which LAMMPS employs for solving the energy and forces of the electrostatic interactions. In this scheme, the point charges of atoms are smoothed using Gaussian charge distributions, and the sum of these distributions is gathered into a regular 3D grid that fills the simulation cell. The charge density on the grid is then transformed into a reciprocal space using fast Fourier transform (FFT). The electric potential of a charge density distribution can be solved using the Poisson equation, and the solution is particularly easy in the reciprocal space. The inverse transform of the solution gives the electric potential in real space.

At a sufficient distance from the atoms, the electric potential generated by the smooth charge distribution is approximately equal to the one generated by the original point charges. The sufficient distance depends on the width of the smoothing Gaussian functions, and we have chosen this width to be narrow enough (standard deviation σ = 0.05 nm) for the calculation of the potential at 0.1 nm away from the atoms with a negligible error compared to the potential of the point charges. From a physical point of view, the Gaussian charge distributions are actually more realistic than point charges, but we preferred the electric potential to be consistent with the one that affects the dynamics of atoms in the MD simulations.

Supplementary Note 1: Orientation of Water Molecules

As explained in the introduction, clinochlore consists of alternating T and B layers, which are positively and negatively charged, respectively. Constructing a simulation model with an even number of layers (the same number of the T and B layers) results in a charge neutral system, but has the downside that an inherent dipole is present (pointing from the negative T towards the positive B layers. Although internal dipoles between the layers are cancelled, a residual charge on the top and bottom layers created a net electric dipole along the [001] direction. As a consequence of this net dipole, the water molecules order themselves far from the surface (several tens of nanometres), not just the expected first few hydration layers (see insets of Supplementary Fig. 5a,b). In order to show this abnormal behaviour in more detail, we computed the orientation of the dipole angle with respect to the surface normal (Supplementary Fig. 5a,b). As can be seen, water in the first few hydration layers is ordered according to the nearby surface charge, but also distant water follows this distribution. We realised that building a simulation structure with an even number of clinochlore layers (even with a simulation that is completely charge neutral) is not appropriate, as it causes this nonphysical behaviour of the water. The only solution is to use an odd number of layers in the clinochlore and terminating the simulated crystal on both free surfaces with either the T or B layers. This has a side-effect that the entire simulated system is now no longer charge neutral (something that is not desirable when using a Particle Mesh Ewald solver for the long-range coulomb forces). The only solution to maintain charge neutrality is to add ions to the solution conveniently mimicking the experimental conditions. As can be seen in Supplementary Fig. 5c,d this ensures that the water in the bulk no longer shows the long-range ordered behaviour. Only a limited number of ions need to be added to counter the residual charge, but typically, we add up to 1 M of NaCl at the initial state with the effective pH determined by the surface charge imbalance. Typical simulated systems used in this study are shown in Supplementary Fig. 6.

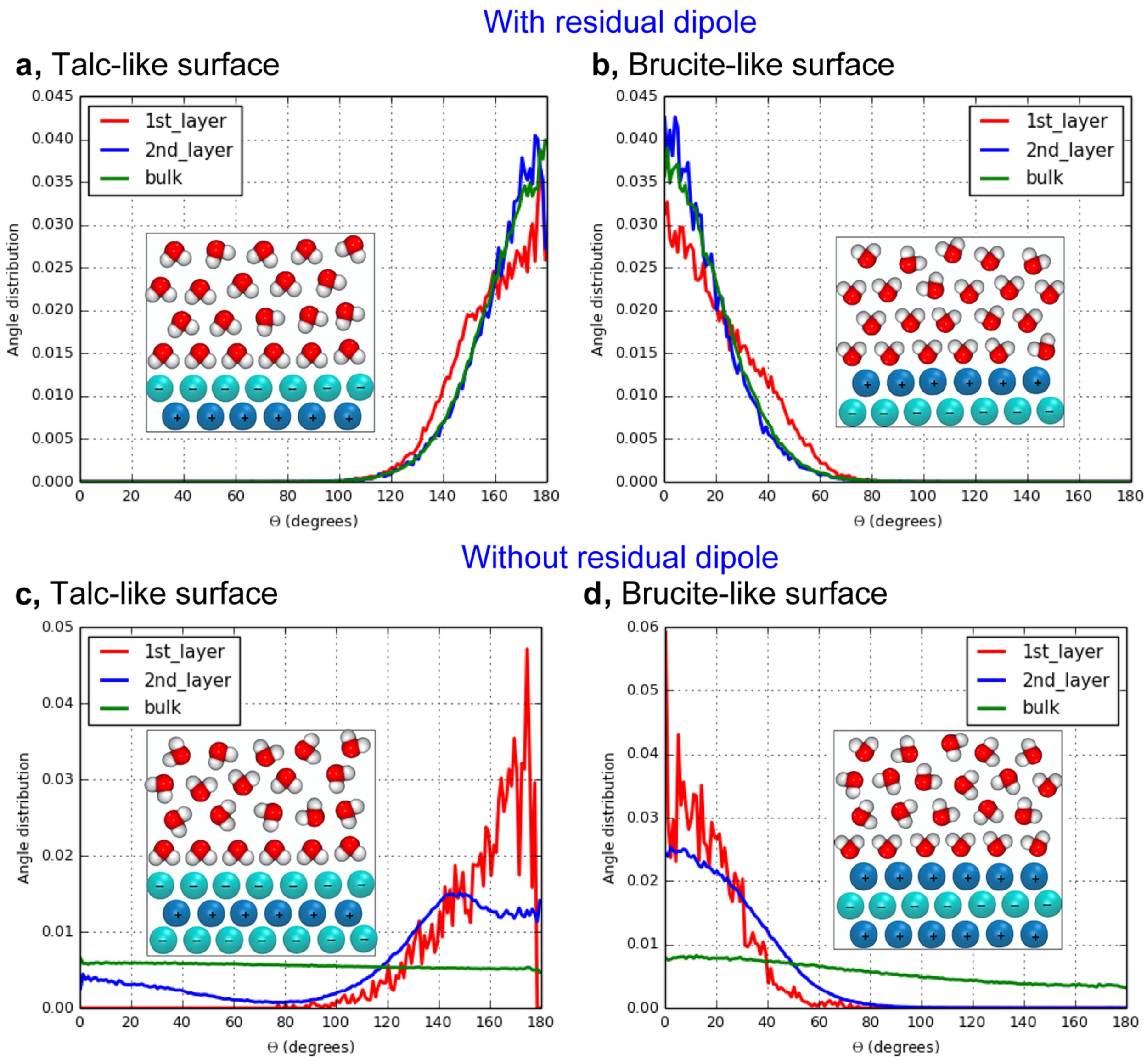

Supplementary Figure 5 | Dipole vector of the water molecules. a,b,c,d, Probability distribution of the dipole vector of the water molecules with respect to the surface normal on the talc-like surface (**a,c**) and brucite-like surface (**b,d**) in the first (red) or second (blue) hydration layer and in the bulk phase (green). Note that 1st and 2nd in the talk-like surface data correspond to the $1st_L$ and $1st_H$, respectively. In **a** and **b,** a residual dipole is present resulting in the nonphysical behaviour of the water far away from the surface (i.e., the water remains structured). Insets are schematic of the surface and water molecules created using VMD[3]. The cyan (or bright blue) spheres represent the talk-like surface and dark blue spheres represent brucite-like surface, red and white atoms are water molecules. This is a simple 2D representation based upon the average trajectories from simulations.

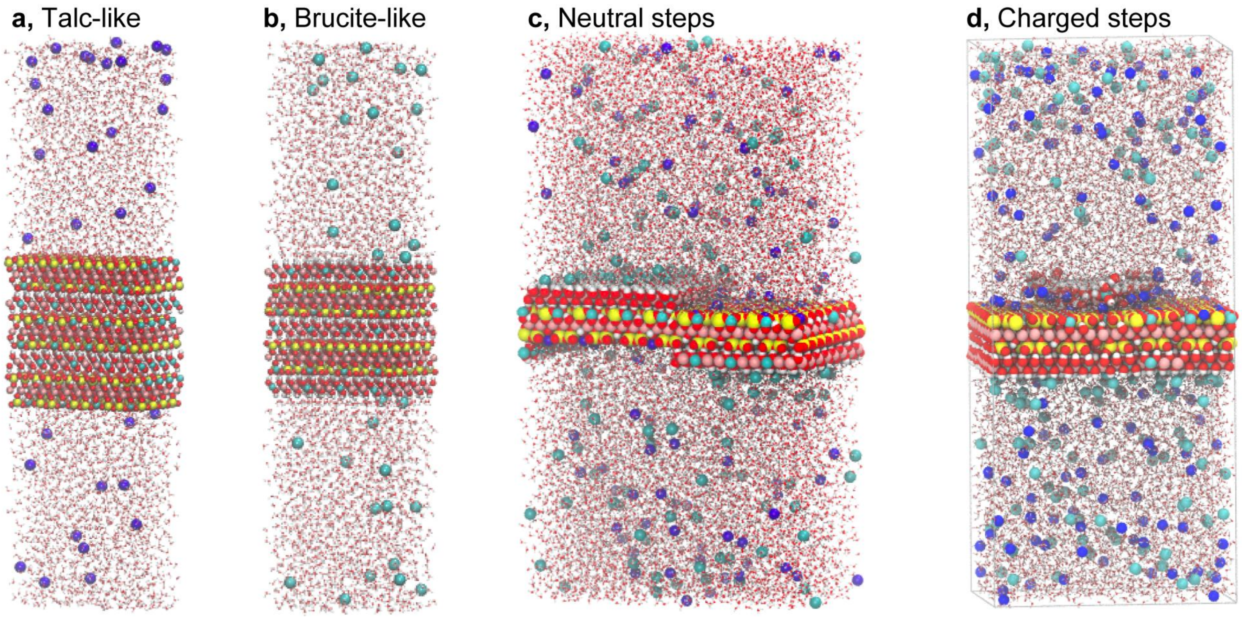

Supplementary Figure 6 | Snapshots from the MD simulations. a,b, Clinochlore crystal with the talc-like (**a**) and brucite-like (**b**) layers exposed on either side, **c,** neutral steps, and **d,** negatively charged triangular step/pit. The water molecules are drawn as red-white lines, whereas the ions are shown in cyan (Cl^{-}) or blue (Na^{+}). The figures were drawn using VMD[3].

In order to investigate in more detail water structuring at different surfaces (T and B), we calculated the average number of hydrogen bonds as a function of the distance from each surface. The number of water-water hydrogen bonds (H-bonds) was calculated by using geometrical criteria[4], where two conditions are fulfilled: (1) distance between H – $O_{acceptor}$ atoms ranges from 1.59 to 2.25Å and (2) angle between O_{donor} – H – $O_{acceptor}$ atoms ranges from 140° to 180° as shown in Supplementary Fig. 7a. The results in Supplementary Fig. 7b,c show clearly that water is more structured close to the surface, where the average number of the H-bonds at the peak is higher than for bulk water. The missing peaks in the first hydration layers, namely the adsorbed first lower layer on T-like surface and the first layer on B-like surface, demonstrate that the water orientation in the first hydration layers is strongly influenced by the interaction with the surface, and only few *water-like* bonds can be seen.

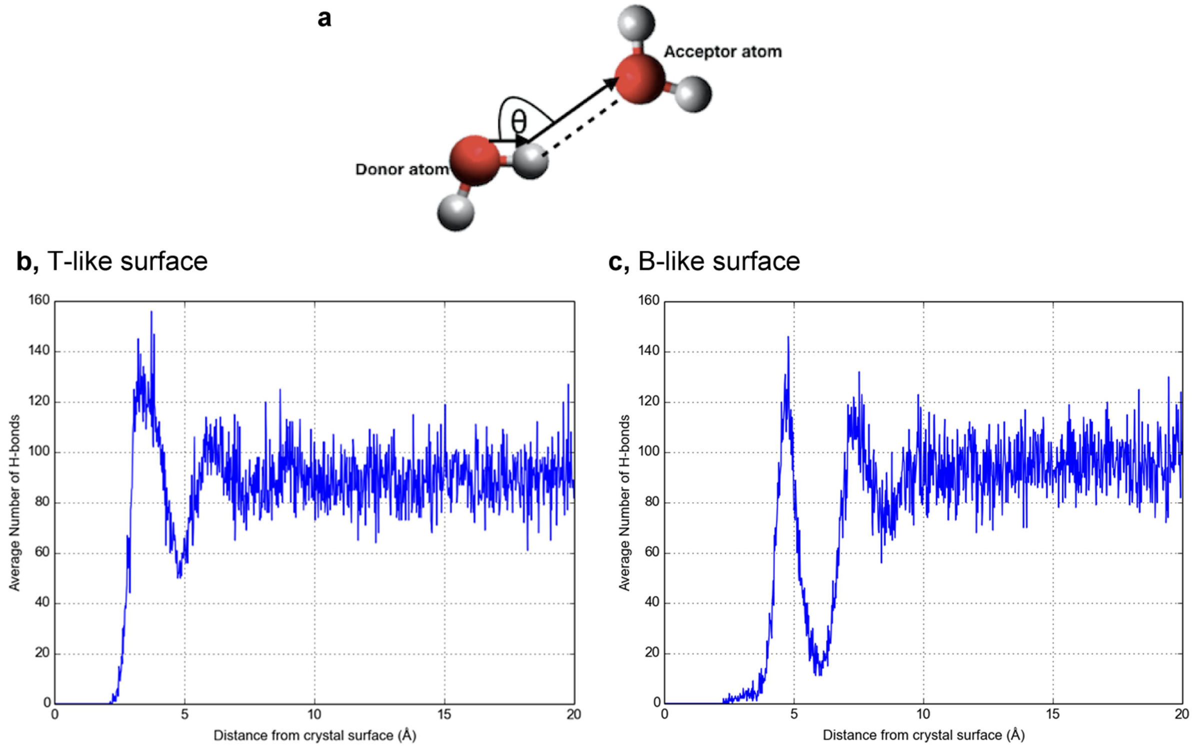

Supplementary Figure 7. Average number of water-water H-bonds as a function of distance from the surface. a, Schematic of the H-bond definition. **b,c,** Average numbers of the H-bonds on talc-like (**b**) and brucite-like (**c**) surfaces.

Supplementary Note 2: Surface Charge Densities on Brucite-Like Regions

In order to estimate the surface charge densities on the B_I and B_{II} regions, we analysed the long-range electric double layer force using the same method as we previously established[5]. We used a sphere and cone model for the tip, and infinite planar models for the B_I and B_{II} surfaces. The effective surface charge density of the SiO_2/Si tip was calculated by the charge-regulation boundary condition. Since the estimation of the surface charge density of the sample sensitively depends on the tip radius, it should be exactly determined during the experiments for quantitative estimation of the surface charge density. However, we can estimate the ratio of the surface charge densities on the B_I and B_{II} regions even without the exact value of the tip radius.

Supplementary Fig. 8 shows the laterally averaged force curves for the B_{II} region from the 3D-FM-AFM experiment. Although oscillatory hydration features similar to those on the B_I region were observed, the amplitude was merely about 10 pN, which was much less than that on the B_I region. We fitted the long-range attractive force by an exponential function with the Debye distance of 0.9 nm. From the Debye length, we assumed the ion concentration as 110 mM, which was slightly higher than the prepared concentration due to the evaporation of water during the experiment. We first calculated the surface charge densities assuming the apparent surface to the outer Helmholtz planes for both surfaces. By assuming the tip radius ranging from 6 to 8 nm, we estimated the charge density ranging from +0.32 to +0.09 C/m^2 and that ranging from +0.09 to +0.04 C/m^2 for the B_I and B_{II} regions, respectively. From the experiment, the apparent surface on the B_{II} region is considered to be the bare surface, and thus we could estimate the surface charge density of the B_{II} region as approximately +0.05 C/m^2 by setting the zero distance to the force maximum corresponding to the first hydration layer.

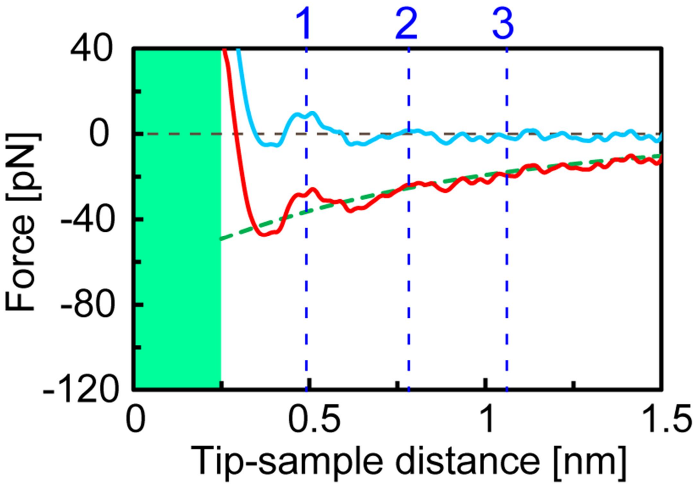

Supplementary Figure 8 | Fitting of electric double layer forces on the B_{II} region. The average force profiles on the B_{II} region were extracted from the 3D force map at 200 randomly selected pixels (red curves). The green area represents the apparent thickness of the B layer (0.25 nm). The broken green and solid light blue curves are the fitted background force and subtracted force curves, respectively. The vertical blue broken lines represent the force maxima in the subtracted force curves.

Supplementary Note 3: Comparison of simulated force data

The experimental and theoretical data in Figs. 2 and 3 have many features in common. However, this similarity is not uncommon[6-8], either for the force or frequency shift data with respect to the simulated density data, although, in principle, we cannot directly compare them[7]. To overcome this problem, we used, as a first approximation, the recently proposed solvent tip approximation (STA) model, which can quickly provide a possible interaction force distribution[9-11] without performing extensive and computationally intensive free energy calculations of systems incorporating the tip[7,12]. The simulated hydration force F is described as

$$F(z) = \frac{k_B T}{\rho(z)} \frac{\mathrm{d}\rho(z)}{\mathrm{d}z},$$

where k_B, T, ρ, and z are the Boltzmann constant, temperature, water density and the distance between a water molecule on the tip and the sample surface, respectively.

We converted the simulated water density to force map data on each terrace region. Supplementary Fig. 9a,b show the simulated force maps in the first and second layers based on the STA model, respectively. The honeycomb-like pattern of the first hydration force on the T region (left side in Supplementary Fig. 9c) is almost similar to the simulated water density in Fig. 2e, although adsorbed waters in the centre of the honeycomb are also visible again. The second hydration layer above the T region shows a more clear dot-like pattern (left side in Supplementary Fig. 9d) which is much more similar to the experimental force map in Fig. 2d than the simulated water density in Fig. 2f. In the case of the B_I region, both the first (right side in Supplementary Fig. 9c) and second (right side in Supplementary Fig. 9d) hydration layers exhibit the same patterns as the corresponding simulated water densities (Fig. 2i,j). Note that all of the colour scale bars are set to the same range because the force values are almost the same order. From the simulated force data, we extracted force maps perpendicular to the clinochlore surface along the lines indicated in Supplementary Fig. 9c,d, On both of these maps, the periodic dot-like patterns are more clearly observed than the water densities in Fig. 2l,n and agree with the experimental results, which means that this model is plausible.

Supplementary Fig. 9e,f show the simulated force curves for the T and B_I regions, respectively. Since the STA model does not take into account the background forces, we compare the simulated and experimental force curves after the subtraction of the background forces (light blue curves in Fig. 3) instead of the original force curves and we found many common features between the simulated force curves and the experimental force curves. They are in good agreement overall except that the magnitude of the hydration force oscillation associated with the second hydration layer on the T region expected from the STA model is smaller than that observed in the experiment. Note that the simulated and experimental force curves after the subtraction on the B region are very much similar. It should also be mentioned that the distances between the force maxima are closer to those in the experimental curves (0.27 nm) rather than those in the 1D water density curves, likely because of the absence of an explicit tip[13].

Regarding the terrace regions, we could obtain consistent results with the water densities. We then compare the counterpart of the perpendicular water density maps in Fig. 5b. Supplementary Fig. 9g shows the simulated perpendicular force map around the step edge. Although we obtained an indistinct hydration force of the second hydration layer, we could not obtain the lower first layer at the intermediate region as seen in the experimental result.

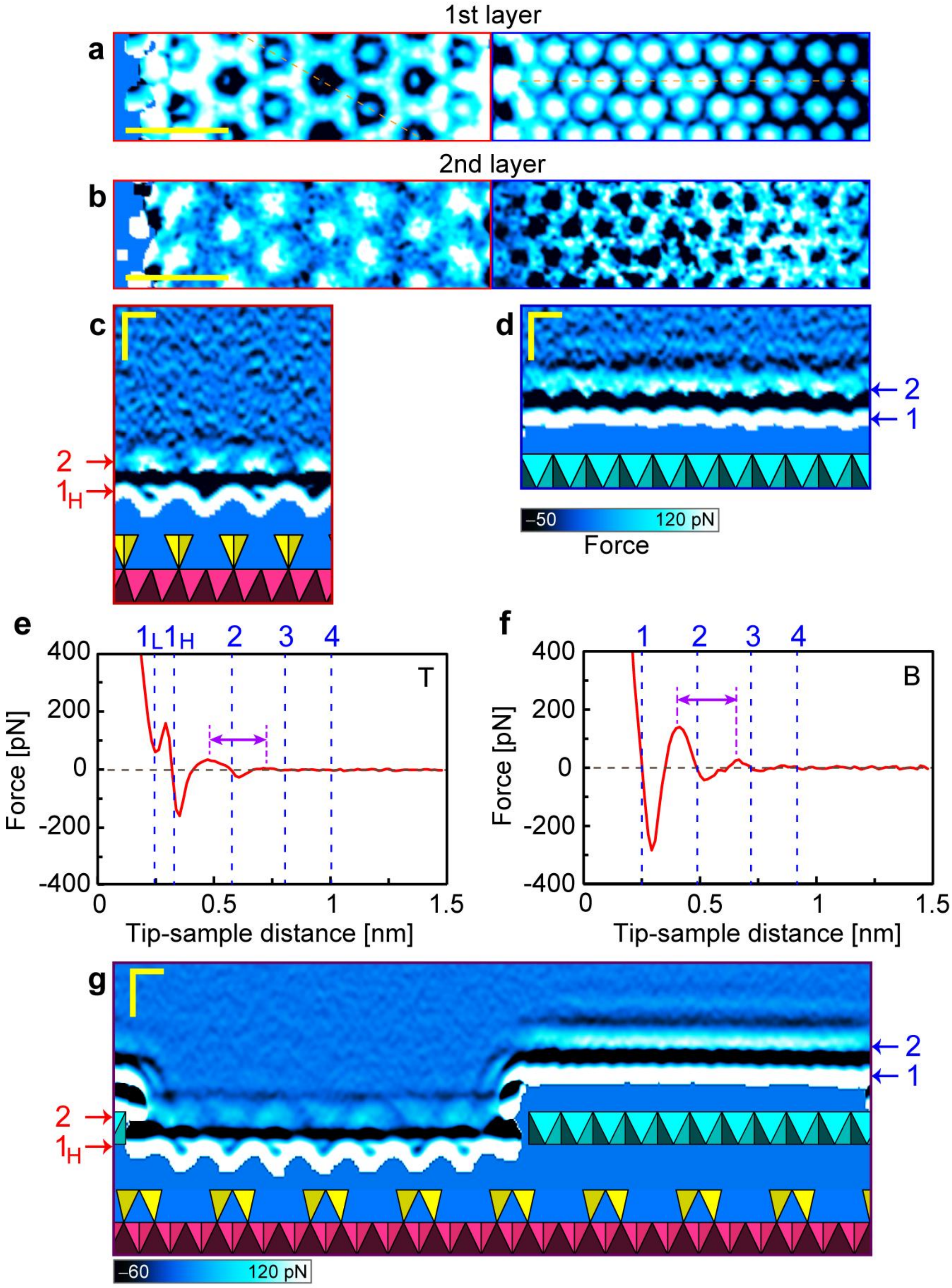

Supplementary Figure 9 | Comparison of simulated force data. a,b, Simulated lateral force maps in 1st layer on the T region and first layer on the B region (**a**) and second layers on the T and B regions (**b**). **c,d,** Theoretical perpendicular 2D force maps along the broken lines in **a** on the T region (**c**) and the B region (**d**). Colour-scale bar at the bottom of **d** was used for **a**–**d**. **e,f,** Simulated force curves calculated by the STA-model from the water densities of Fig. 3c,d, respectively. **g,** Simulated perpendicular force maps averaged along the [001] direction, where the arrows indicate the height of the lateral force maps in **a** and **b**. Scale bars, 1 nm (**a,b**) and 0.3 nm (**c,d,g**).

Supplementary Note 4: Reference measurement in ultra-pure water

We conducted the hydration measurement on muscovite mica (001) surface in ultra-pure water in order to deny the possibility that the observed "hydration structures" mainly reflect the ion distributions. The actual experimental solution may have not been an ultra-pure grade due to any contaminants from air as well as the cations dissolved from mica surface during the experiment. We successfully obtained atomic resolution topographic and hydration images shown in Supplementary Fig. 10. The surface is not as flat as the T-layer region observed in 100 mM KCl solution, and it shows nanometre-scale undulation features similar to those we previously reported[14]. This may be due to that the orientations of water molecules are stabilised by the adsorbed ions and/or the charged surface adsorbs contaminants.

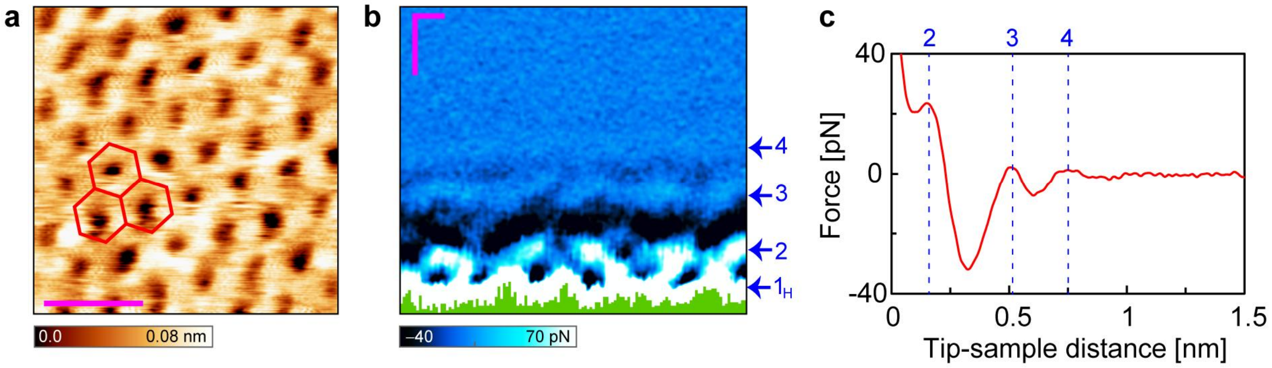

Supplementary Figure 10 | Hydration measurement in ultra-pure water. a,b, Topographic (**a**) and 2D force map (**b**) images of the muscovite mica (001) surface. **c,** Average force profile extracted from 2D force map in **b**. Scale bars, 1 nm (**a,b**) and 0.3 nm (**c**).

Supplementary Note 5: Bacteriorhodopsin Membrane Suspended on Clinochlore

We deposited a bacteriorhodopsin (bR) membrane on the clinochlore (001) surface to demonstrate that the substrate is useful as an experimental platform for exploring the structures and properties of biological molecules. Supplementary Fig. 11a shows an FM-AFM image of the bR membrane on the clinochlore (001) surface obtained in a 10 mM phosphate buffer solution. Since the membrane was suspended on the B-layers, the membrane is partly isolated from the substrate. Therefore, it is useful to study the biological functions of the transmembrane proteins such as bR. A possible experimental scheme is shown in Supplementary Fig. 11b.

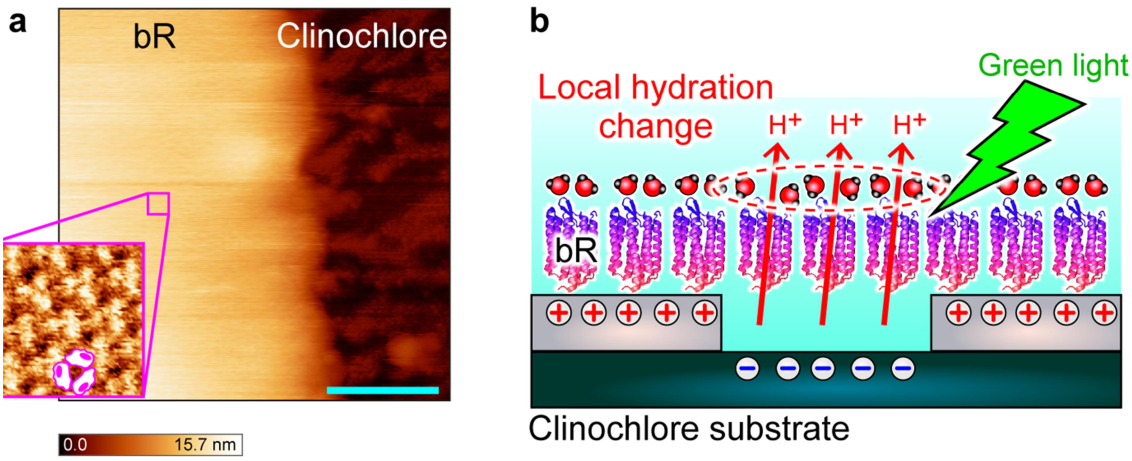

Supplementary Figure 11 | Bacteriorhodopsin membrane suspended on clinochlore. **a,** Topographic image of bR membrane adsorbed on the clinochlore (001) surface, which exhibits both the membrane and bare clinochlore surfaces, and molecular-scale image of bR molecules (inset). **b,** Schematic of an experiment for detecting the structural changes in the hydration structures of the bR molecules in the membrane suspended between the clinochlore (001) terraces upon irradiation of green light[15,16]. Scale bar, 50 nm (**a**).

Supplementary Note 6: Charge States of Brucite-like layer at Step Edges

Upon cleavage of clinochlore, the charge states of the B layer at the step edges vary depending on whether the cut is along the armchair (U–D) or zigzag (L–R) line in Supplementary Fig. 12. Supplementary Fig. 12a shows models for the armchair cut-direction, of which both the separated edges are neutral. Along these steps the metal cations and the oxygen atoms are all on the same line (no oxygen atoms are protruding), and the total charge along the step is zero, but locally there are charge differences. Supplementary Fig. 12b shows models for the zigzag cut-direction. While only the edge with exposed oxygen atoms, which are part of the fully coordinated sphere of metal ions, is negatively charged, the other edge with Al^{3+} or Mg^{2+} cations is positively charged. These cations easily dissolve into a solution to form a different configuration of the negatively charged edge. The step edges with edge-exposed octahedral are more stable than those with corner-exposed octahedral[15]. The preferential dissolution of the unstable edges eventually transform to the stable edges. Among these edges, the negatively charged edges are mostly seen in the experiments.

As discussed in the main text, alongside the neutral step ("Neutral edges" model in Supplementary Fig. 12b), we have also simulated a triangular step structure ("Stable negative edge" model in Supplementary Fig. 12b) as a potential candidate for the real step edge structure in the experiments. Our simulation model consists of a triangular step on the top of T layer and the triangular pit on the bottom (see Supplementary Fig 6d). In order to maintain a charge neutral system, the triangular pit consists of exposed metal ions and was positively charged ("Positive edge" model in Supplementary Fig. 12b). Simulations showed that this triangle step was not stable, even with fixed Al atoms at the corners, as significant hydroxyl groups detach from the surface. We also noticed that step edges with edge-exposed octahedral are more stable than those with corner-exposed octahedral[17]. A significant increase in stability was observed when introducing a nonbonded three-body harmonic potential energy term for Mg-O-H interactions[18]. However, this didn't prevent a few hydroxyl groups from still leaving the surface.

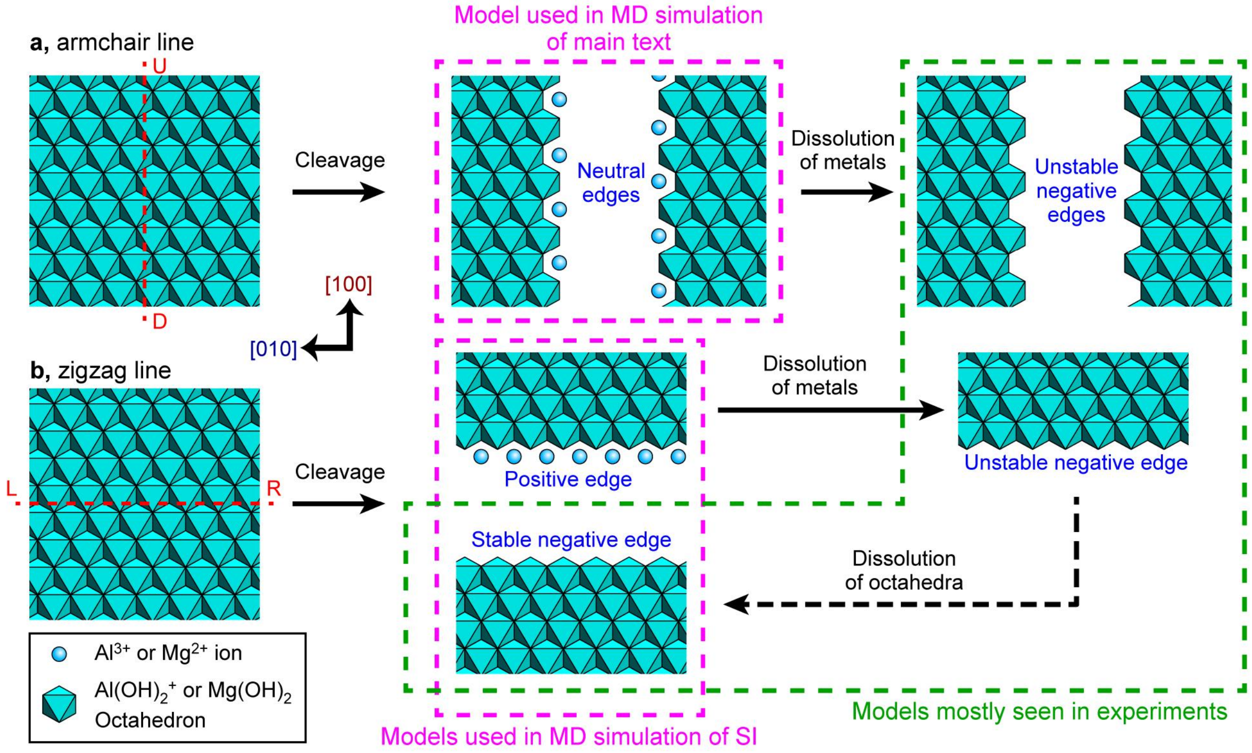

Supplementary Figure 12 | Schematic models of brucite-like layer. a,b, Models showing neutral edges (**a**) and oppositely charged edges (**b**) before and after cleavage.

Simulation results for charged step edges are summarised in Supplementary Fig. 13. In this case, all the three sides of the triangle have the stable negatively charged edges (Supplementary Fig. 13a). On the T layer, the honeycomb-like water and dot-like ion distributions being same as the data without the B layer can be seen (Supplementary Fig. 13b,c). Unlike the case of the neutral edges, Na ions are clearly attracted to the sides of the step edges (Supplementary Fig. 13e) due to its composition (exposed oxygens) and charge, which partially neutralise the negative oxygen charges. This also affects the water ordering in the vicinities of the step edges (Supplementary Fig. 13d) due to the electrostatic interactions with ions. On the B layer, the water oxygen density show a clear dot-like structure being same as the data without the T layer, while the ion density does not show the clear honeycomb-like distribution (Supplementary Fig. 13f,g). Furthermore, unlike the neutral edges, significant difference of the water and ion densities was not observed around the step edges. In order to ascertain the cause for these discrepancies between the neutral and charged step edges, we analysed the electric potential shown in Supplementary Fig. 13f. Electric potential shows significantly decrease as going from the island terrace to the peripherals as also observed in the neutral edges. However, we noticed that the electric potential in this height on the B layer is not positive but negative because the size of the B layer island was not large enough to fully exclude the influence from the negative potential from the T layer. The negative potential on the B layer reduced the chlorine ion density, which deteriorates the honeycomb-like distribution and causes the depletion of ions around step edges that were clear in the neutral step model. Although we realised that a much larger triangular island was required for examining the edge effect, the instabilities at the step edge prevented further enlargement using the existing force fields. Moreover, the instabilities at the step edge also complicated the interpretation, and therefore we focused only on the neutral stable edge structure in the main text.

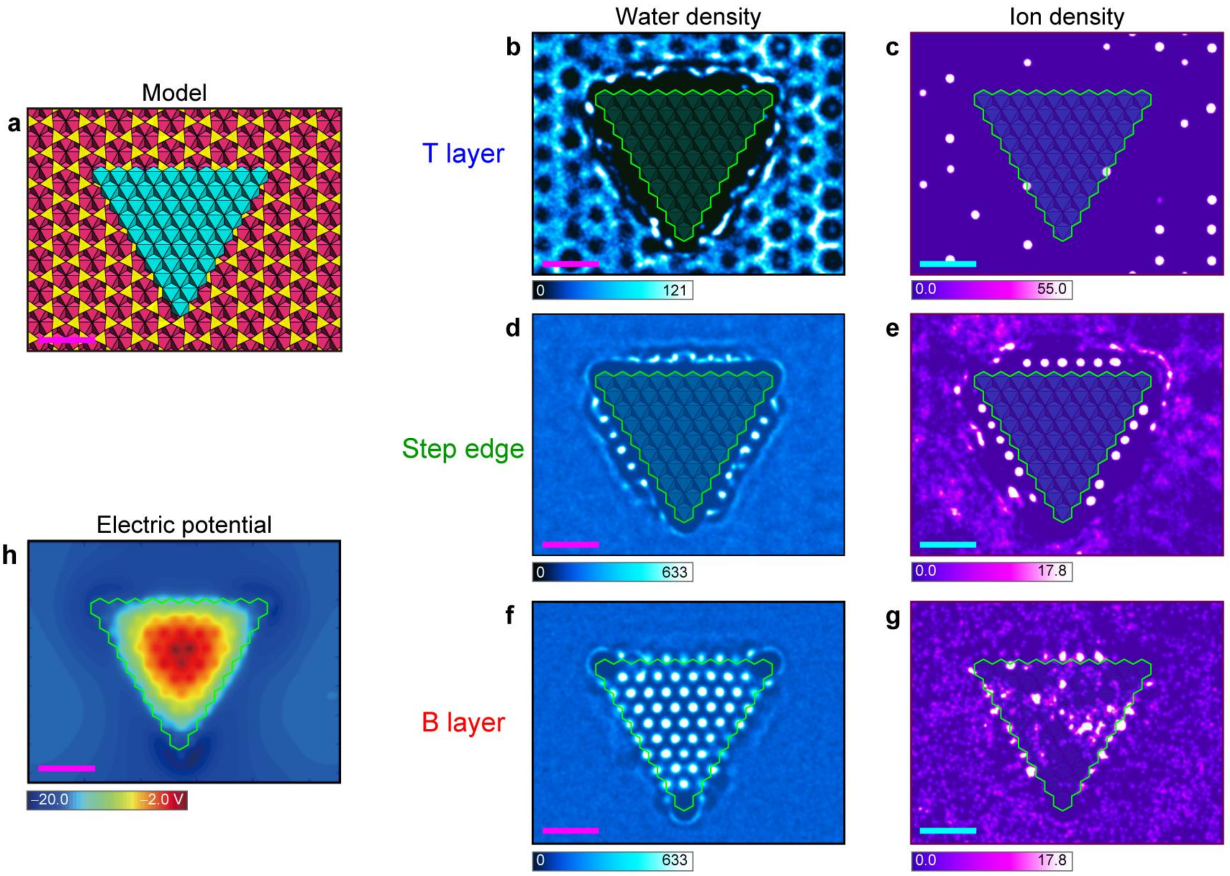

Supplementary Figure 13 | 2D water oxygen and ions densities at the triangular step edge. a, Structural model used in the MD simulation. **b-g,** Normalised Water (oxygen) (**b,d,f**) and ion (**c,e,g**) densities at the same vertical positions as the 1st hydration layer on the T layer (**b,c**), the metal ions in the B layer (**d,e**), and the 1st hydration layer on the B layer (**f,g**). **h,** Electric potential 0.1 nm above the outermost oxygen atoms of the B region. Scale bars, 1 nm (**a-h**).

Supplementary References

1. Valdrè, G., Moro, D. & Ulian, G. Nucleotides, RNA and DNA selective adsorption on atomic-flat Mg-Al-hydroxysilicate substrates. *Micro Nano Lett.* **6**, 922-926 (2011).
2. Phillips, T. L., Loveless, J. K. & Bailey, S. W. Cr^{3+} coordination in chlorites - structural study of 10 chromian chlorites. *Am. Mineral.* **65**, 112-122 (1980).
3. Humphrey, W., Dalke, A. & Schulten, K. VMD: Visual molecular dynamics. *J. Mol. Graphics* **14**, 33-38 (1996).
4. Kumar, R., Schmidt, J. R. & Skinner, J. L. Hydrogen bonding definitions and dynamics in liquid water. *J. Chem. Phys.* **126**, 204107 (2007).
5. Umeda, K., Kobayashi, K., Oyabu, N., Matsushige, K. & Yamada, H. Molecular-scale quantitative charge density measurement of biological molecule by frequency modulation atomic force microscopy in aqueous solutions. *Nanotechnology* **26**, 285103 (2015).
6. Kobayashi, K. *et al.* Visualization of hydration layers on muscovite mica in aqueous solution by frequency-modulation atomic force microscopy. *J. Chem. Phys.* **138**, 184704 (2013).
7. Fukuma, T. *et al.* Mechanism of atomic force microscopy imaging of three-dimensional hydration structures at a solid-liquid interface. *Phys. Rev. B* **92**, 155412 (2015).
8. Spijker, P. *et al.* Understanding the Interface of Liquids with an Organic Crystal Surface from Atonnistic Simulations and AFM Experiments. *J. Phys. Chem. C* **118**, 2058-2066 (2014).
9. Watkins, M. & Reischl, B. A simple approximation for forces exerted on an AFM tip in liquid. *J. Chem. Phys.* **138**, 154703 (2013).
10. Amano, K., Suzuki, K., Fukuma, T., Takahashi, O. & Onishi, H. The relationship between local liquid density and force applied on a tip of atomic force microscope: A theoretical analysis for simple liquids. *J. Chem. Phys.* **139**, 224710 (2013).
11. Miyazawa, K. *et al.* A relationship between three-dimensional surface hydration structures and force distribution measured by atomic force microscopy. *Nanoscale* **8**, 7334-7342 (2016).
12. Reischl, B., Watkins, M. & Foster, A. S. Free Energy Approaches for Modeling Atomic Force Microscopy in Liquids. *J. Chem. Theory Comput.* **9**, 600-608 (2013).
13. Asakawa, H., Yoshioka, S., Nishimura, K. & Fukuma, T. Spatial Distribution of Lipid Headgroups and Water Molecules at Membrane/Water Interfaces Visualized by Three-Dimensional Scanning Force Microscopy. *ACS Nano* **6**, 9013-9020 (2012).
14. Fukuma, T., Kobayashi, K., Matsushige, K. & Yamada, H. True atomic resolution in liquid by frequency-modulation atomic force microscopy. *Appl. Phys. Lett.* **87**, 034101 (2005).
15. Kimura, K. *et al.* Visualizing water molecule distribution by atomic force microscopy. *J. Chem. Phys.* **132**, 194705 (2010).
16. Shibata, M., Yamashita, H., Uchihashi, T., Kandori, H. & Ando, T. High-speed atomic force microscopy shows dynamic molecular processes in photoactivated bacteriorhodopsin. *Nature Nanotech.* **5**, 208-212 (2010).
17. Gazzè, S. A., Stack, A. G., Ragnarsdottir, K. V. & McMaster, T. J. Chlorite topography and dissolution of the interlayer studied with atomic force microscopy. *Am. Mineral.* **99**, 128-138 (2014).
18. Zeitler, T. R., Greathouse, J. A., Gale, J. D. & Cygan, R. T. Vibrational Analysis of Brucite Surfaces and the Development of an Improved Force Field for Molecular Simulation of Interfaces. *J. Phys. Chem. C* **118**, 7946-7953 (2014).
