# Supplementary material for: Atomic-resolution three-dimensional hydration structures on a heterogeneously charged surface
Source: Nat Commun. 2017 Dec 13;8:2111. doi: 10.1038/s41467-017-01896-4 (PMC5727385; doi:10.1038/s41467-017-01896-4)
Supplement: Supplementary file 2 — Description of Additional Supplementary Files [file 41467_2017_1896_MOESM2_ESM.pdf]

Description of Additional Supplementary Files

File Name: Supplementary Movie 1
Description: **Crystal structure of clinochlore.** Polyhedral representation of the clinochlore crystal drawn using VESTA.

File Name: Supplementary Movie 2
Description: **Full 3D-force map obtained by 3D-FM-AFM.** 3D-force map which was obtained on an area including the T, B_{II} and B_{I} regions. Bright and dark colour areas correspond to the repulsive and attractive forces, respectively.

File Name: Supplementary Movie 3
Description: **Full 3D distributions of waters and ions calculated by MD simulation.** Water and ion densities around a neutral clinochlore step. Bright and dark colour areas correspond to the higher and lower densities, respectively.
